# First Fossil Record of *Trichomanes sensu lato* (Hymenophyllaceae) from the Mid-Cretaceous Kachin Amber, Myanmar

**DOI:** 10.3390/life13081709

**Published:** 2023-08-09

**Authors:** Ya Li, Atsushi Ebihara, Natalya Nosova, Zhen-Zhen Tan, Yi-Ming Cui

**Affiliations:** 1 State Key Laboratory of Palaeobiology and Stratigraphy, Nanjing Institute of Geology and Palaeontology, Chinese Academy of Sciences, Nanjing 210008, China; 2 Department of Botany, National Museum of Nature and Science, 4-1-1 Amakubo, Tsukuba 305-0005, Japan; ebihara@kahaku.go.jp; 3 Komarov Botanical Institute of the Russian Academy of Sciences, Prof. Popova Str. 2, Saint Petersburg 197376, Russia; natanosova@gmail.com; 4 College of Agro-Grassland Science, Nanjing Agricultural University, Nanjing 210095, China; tanzhenzi2008@163.com; 5 Lushan Botanical Garden, Chinese Academy of Sciences, Jiujiang 332900, China

**Keywords:** epiphyte, filmy fern, *Hymenophyllites*, Mesozoic, trichomanoid

## Abstract

Hymenophyllaceae (filmy ferns), with ca. 430 species, are the most species-rich family of early diverging leptosporangiate ferns but have a poor fossil record dating back to the Late Triassic period. Traditionally, Hymenophyllaceae comprise two species-rich genera or clades: *Hymenophyllum* (hymenophylloids) and *Trichomanes sensu lato* (*s.l.*) (trichomanoids). Unequivocal fossils of *Hymenophyllum* have been reported from the Early Cretaceous of central Mongolia and the early Eocene of Okanogan Highlands, Washington, USA. However, despite being a highly diversified lineage with an estimated 184 extant species, *Trichomanes s.l.* lack a definitive fossil record, which severely affects the reliability of the molecular dating of this group. Here, we report the first unequivocal fossil record of *Trichomanes s.l.* as *T*. *angustum* comb. nov. on the basis of fertile material with tubular involucres and long exserted receptacles from the mid-Cretaceous Kachin amber, Myanmar. This species was previously tentatively assigned to *Hymenophyllites* due to a lack of fertile evidence. Inferred to be an epiphytic fern, *T*. *angustum* further enriches the species diversity of the epiphytic palaeocommunities in the mid-Cretaceous Kachin amber, which are mainly composed of Porellalean leafy liverworts and Dicranalean and Hypnodendralean mosses. Fossil records indicate that Hymenophyllaceae probably originated in the tropical Pangea at the latest in the Triassic when all continents were coalesced into a single landmass and had already accumulated some notable diversity in low-middle latitude areas of Laurasia by the mid-Cretaceous period.

## 1. Introduction

Hymenophyllaceae (filmy ferns) include ca. 430 extant species [1] and are the largest family of early diverging leptosporangiate ferns [1,2,3,4,5,6,7,8]. Hymenophyllaceae are predominantly distributed in moist mossy forests in tropical mountains and south-temperate areas [9] and are mainly composed of epiphytic ferns but also include some terrestrial and climbing species [10,11]. Hymenophyllaceae are mainly characterized by a reduction of the leaf lamina into usually one-cell-layer thick lamina between the veins with the absence of stomata, and the presence of marginal indusiate sori, sporangia with an oblique annulus, and trilete spores [4,9,12,13,14,15,16]. The phylogenetic position of Hymenophyllaceae was not well resolved [1,4] until 2018. The phylogenomic analyses using transcriptome sequencing data strongly support the idea that Hymenophyllaceae and Gleicheniaceae formed a monophyletic clade sister to Dipteridaceae [7,8]. This phylogenetic relationship was also supported by sporangia characters [8]. Traditionally, Hymenophyllaceae comprise two species-rich genera or clades: *Hymenophyllum* Sm. (hymenophylloid) and *Trichomanes* L. *sensu lato* (*s.l.*) (trichomanoid) [13,16,17,18,19]. *Hymenophyllum* has bivalvate involucres (indusia) with receptacles usually included within the valves or rarely and shortly exserted, while *Trichomanes s.l.* is characterized morphologically by its tubular or campanulate involucres and hair-like receptacles that are usually long-exserted [16].

Although it is clear that filmy ferns are ancient lineages according to their near-basal phylogenetic position in early-diverging leptosporangiate ferns [1,5,6,7,8,20], reliable fossils of this family are scarce due to low fossilization potential of the delicate, membranous laminae [9,11,21,22,23,24], hampering detailed reconstructions of the origins of their extant diversity. The earliest convincing fossils of the Hymenophyllaceae were reported as *Hopetedia praetermissa* Axsmith et al. from the Late Triassic (Carnian) Pekin Formation of North Carolina, USA [21]. *Hopetedia* Axsmith et al. display a character state mosaic of the two extant genera, namely *Trichomanes*-like funnel-shaped indusia and *Hymenophyllum*-like included receptacles [21]. *Eogonocormus* Deng from the Lower Cretaceous of northeastern China is a small thalloid plant with creeping rhizomes and marginal sori with in situ spores, borne on fanlike pinnule lobes, which indicate convincingly that this genus belongs to the Hymenophyllaceae [25]. Reliable filmy fern fossils also include *Hymenophyllites* H.R.Goeppert from the mid-Cretaceous of Kazakhstan and Myanmar [26,27] and *Acrostichopteris* Fontaine from the Early Cretaceous of Spain, China and USA [28,29,30,31]. However, it is uncertain with respect to the affinities between the above-mentioned fossil genera and the extant genera of Hymenophyllaceae. The earliest fossils of *Hymenophyllum* have been described as *H*. *iwatsukii* Herrera et al. based on abundant and exceptionally well-preserved lignified material from the Early Cretaceous (Aptian–Albian) of Mongolia [11]. *Hymenophyllum axsmithii* Pigg et al. was described from the early Eocene Okanogan Highlands, Washington, USA [24]. However, despite being a highly diversified lineage with an estimated 184 extant species [1], *Trichomanes s.l.* lack a definitive fossil record to calibrate its internal nodes, which severely affects the reliability and the dating of this group of ferns.

Recently, Li et al. [27] described some sterile lamina fragments from the mid-Cretaceous Kachin amber, Myanmar, as three new fossil species of *Hymenophyllites*, including *H*. *angustus* Y.Li et Y.-D.Wang, *H*. *kachinensis* Y.Li et Y.-D.Wang, and *H*. *setosus* Y.Li et Y.-D.Wang. In the present paper, we transfer *H*. *angustus* to *Trichomanes s.l.* based on newly found fertile material from Kachin amber, Myanmar. *Trichomanes angustum* comb. nov. displays typical trichomanoid characters, namely tubular involucres and long exserted receptacles, and thus represents the first unequivocal fossil trichomanoid filmy fern known so far. We also discuss its palaeoecological implications and infer the palaeogeographic history of Hymenophyllaceae based on fossil records.

## 2. Materials and Methods

Kachin amber originates from several amber mines about 20 km southwest of the village of Tanai in the Hukuang Valley of Kachin State, northern Myanmar [32,33]. Kachin amber deposits are currently the most important source of Cretaceous amber-preserved paleobiota [32,34] and yield a large number of plant and animal inclusions [35,36]. The age of Kachin amber is regarded as the late Albian–early Cenomanian, based on the evidence of the ammonite *Puzosia* Matsumoto and palynomorphs [37,38]. The U-Pb dating of zircons suggests the earliest Cenomanian age (98.79 ± 0.62 Ma) for the amber-bearing horizon of Kachin amber [34].

The fossils studied here include two pieces of Kachin amber from Myanmar and are housed at the Collection Department of Nanjing Institute of Geology and Palaeontology, Chinese Academy of Sciences, Nanjing, China, under accession numbers PB200744 and PB201715. Concerning the recent conflicts in Myanmar [39], we declare that we followed the recommendations by Haug et al. [40]. All Kachin amber pieces mentioned in this study were acquired in compliance with the laws of Myanmar and China, including Myanmar’s import and export regulations of jewelry and China’s fossil law. The extant specimens were deposited at the National Museum of Nature and Science, Tsukuba, Japan.

The amber inclusions were studied under a ZEISS Axio Zoom.V16 microscope (Carl Zeiss AG, Oberkochen, Germany) equipped with a high-resolution digital camera (Axiocam 512 color, Carl Zeiss AG, Oberkochen, Germany). Incident and transmitted lights were used simultaneously for photography. All images were digitally stacked photomicrographic composites of ca. 20–50 individual focal planes using the software package ZEN 2.3 pro for a better illustration of the three-dimensional inclusions. The palaeocoordinates of fossil sites were converted from their extant coordinates using PointTracker v4c software and plotted on individual palaeogeographic maps (Mollweide projection) at three time points, i.e., Late Triassic (220 Ma), mid-Cretaceous (120 Ma), and early Eocene (50 Ma), using ArcView GIS 3.2 software. The terminology for the description of the present fern fossils follows Axsmith et al. [21] and Liu et al. [14].

## 3. Results

### Systematic Paleontology

**Order:** Hymenophyllales A.B.Frank

**Family:** Hymenophyllaceae Mart.

**Genus:** *Trichomanes* L. sensu lato

**Species:** *Trichomanes angustum* (Y.Li et Y.-D.Wang) Y.Li et Ebihara, comb. nov.

**Figure 1:**
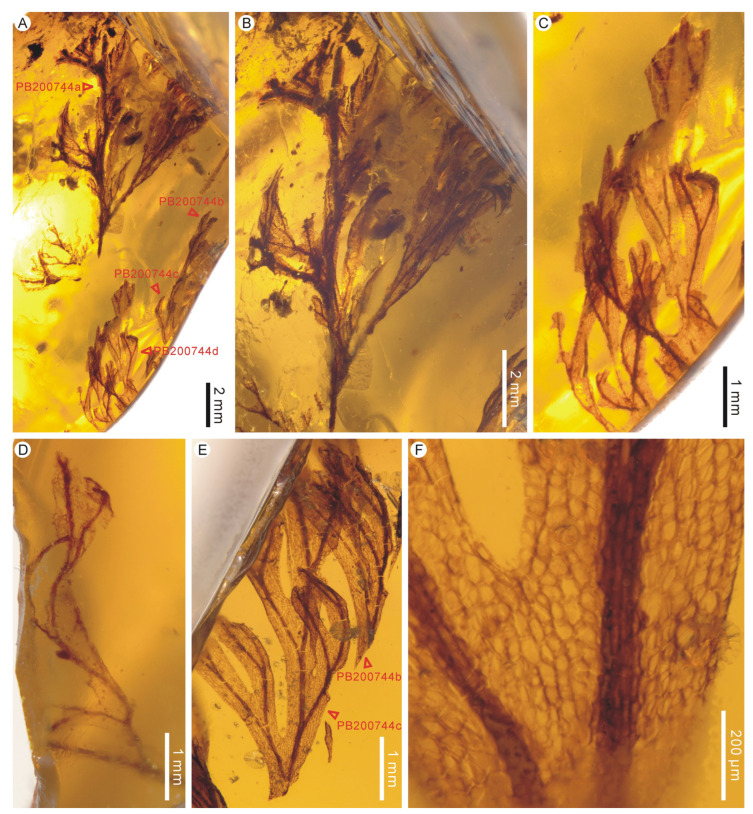
Sterile lamina fragments of *Trichomanes angustum* comb. nov. from mid-Cretaceous Kachin amber. PB200744. (**A**) Lateral view of the amber showing four lamina fragments. PB200744a–d. (**B**) A tripinnatifid lamina fragment showing the rachis winged only at the apex of lamina. Holotype PB200744a. (**C**) A lamina fragment. PB200744d. (**D**) A lamina fragment. PB200744h. (**E**) Lamina fragments. PB200744b, c. (**F**) Enlargement of lamina showing cell shape and arrangement. PB200744c.

**Synonym:** *Hymenophyllites angustus* Y.Li et Y.-D.Wang [27] (pp. 3–5, Figure 1)

**Holotype and Paratypes:** PB200744a and PB200744b–h

**New specimens:** PB201715a, b

**Age:** Late Albian–early Cenomanian, mid-Cretaceous.

**Type locality:** Amber mines southwest of the village of Tanai ca. 105 km north of Myitkyina in Kachin State, northern Myanmar.

**Repository:** Collection Department of Nanjing Institute of Geology and Paleontology, Chinese Academy of Sciences, Nanjing, China.

**Specific diagnosis:** Leaf fragments tripinnate, glabrous. Pinnae leaf fragments closely spaced and alternate. Pinnules of leaf fragments are closely spaced, anadromous, distinctly divided into simple or forked segments. Segments dichotomized 0–3 times to form one to several ultimate lobes. Lobes narrow, slightly elongated, (0.1–) 0.3–0.4 mm wide, entire-margined, with a single veinlet. Lamina venation is anadromous without false veinlets. Differentiated marginal elongated cells are absent. Sori paratactic, borne at the apex of the first arising lobes of the pinnule or its segments. Involucres tubular, ca. 3–5 times as long as wide. Involucre mouths are usually non-dilated. Receptacles are filiform and long exserted.

**Figure 2:**
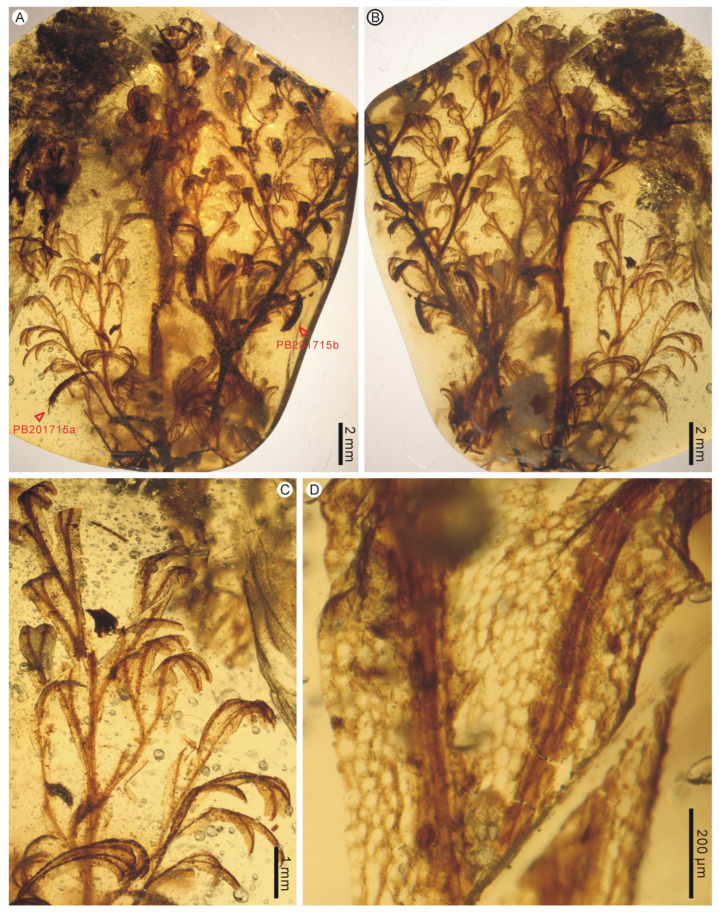
Fertile lamina fragments of *Trichomanes angustum* comb. nov. and its sterile lamina portions from the mid-Cretaceous Kachin amber. PB201715. (**A**,**B**) Two fertile lamina fragments in adaxial and abaxial views. PB201715a, b. (**C**) Apical portion of a sterile pinna. PB201715a. (**D**) Enlargement of lamina showing cell shape and arrangement. PB201715a.

**Figure 3:**
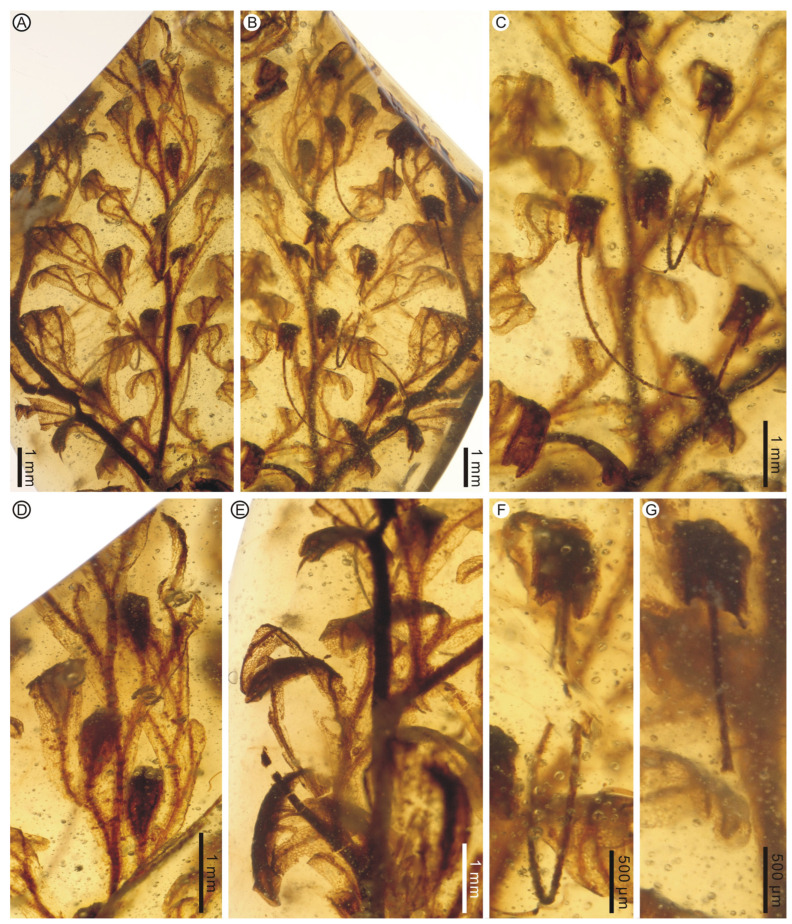
Fertile pinnae and sori of *Trichomanes angustum* comb. nov. from mid-Cretaceous Kachin amber. PB201715b. (**A**,**B**) A fertile pinna in abaxial and adaxial views. (**C**) Enlargement of pinna in adaxial view showing apical portions of sori. (**D**) Enlargement of pinna in abaxial view showing basal portions of sori. (**E**) Several sori in lateral view. (**F**,**G**) Apical portions of two sori showing non-dilated involucre mouths and filiform, long exserted receptacles.

**Figure 4:**
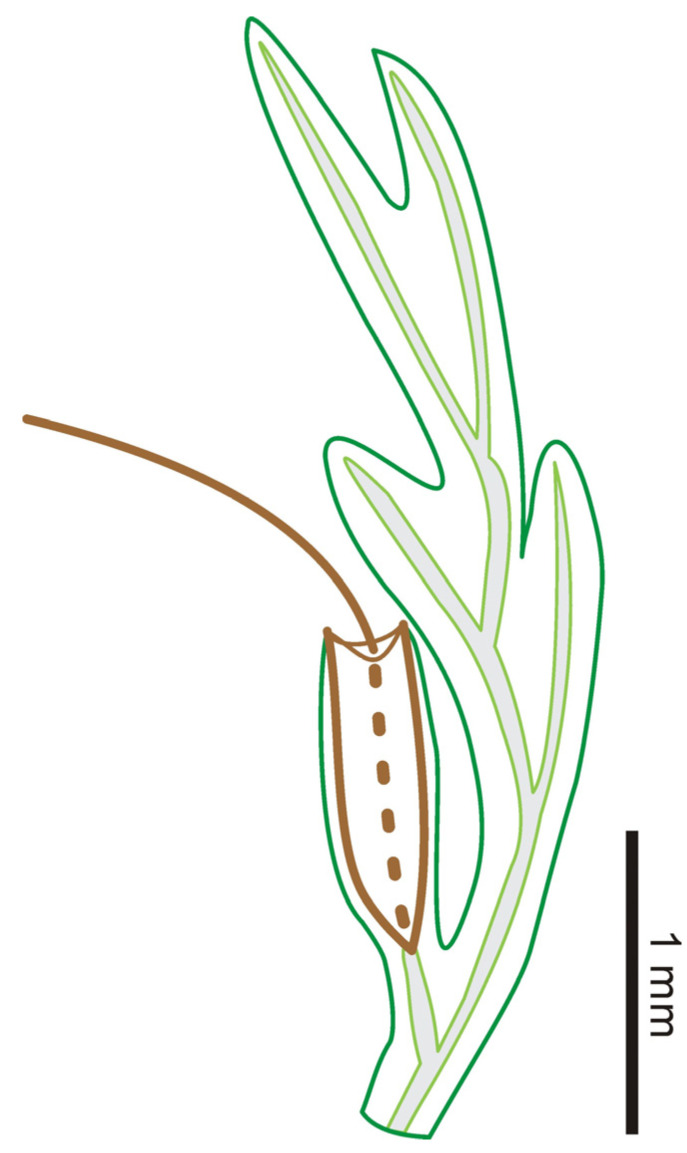
Schematic drawing of fertile pinnule of *Trichomanes angustum* comb. nov.

**Description:** The two large leaf fragments (PB200744a and PB201715b) are likely lamina portions, tripinnate, and are up to ca. 2.1 cm long (Figure 1A,B and Figure 2A,B). However, it is also possible that they actually stem from single large lateral pinnae, as stripes have not been found yet. Lamina is membranaceous, one cell layer thick between the veins, and glabrous (Figure 1C–F and Figure 2C,D). The lamina rachis (also possibly pinna costa) is not winged or winged only at the apex, glabrous (Figure 1B and Figure 2A,B). The pinnae of leaf fragments are at least in 3 pairs, closely spaced, alternate, spread, triangular-ovate, up to 1.2 cm long (Figure 1A,B and Figure 2A,B). The pinnules of leaf fragments are closely spaced, anadromous, up to 0.8 cm long, often with the most proximal ones overlapping the rachis (Figure 1A,B and Figure 2A,B). The pinnules are distinctly divided into segments which are further dichotomized 0–3 times to form simple to several ultimate lobes (Figure 1A,B and Figure 2A–C). The lobes are flat, narrow, slightly elongated, (0.1–) 0.3–0.4 mm wide, entire-margined, with acute, obtuse, truncate to retuse apices (Figure 1C–E, Figure 2C, Figure 3A,B and Figure 4). Each lobe is vascularized by a single veinlet. Lamina venation is anadromous and dichotomous to form more or less zigzag costae and costules. False veinlets are absent. Laminar cells are more than 3 rows between the midrib and margins, polygonal, isodiametrical to slightly elongated with thin and straight cell walls, 25–88 μm long and 14–66 μm wide outside the venation area; however, they are also elongated fusiform to rectangular inside the venation area (Figure 1F and Figure 2D). Differentiated marginal elongated cells are absent. Stomata are absent. Sori are paratactic, borne at the apex of the first arising lobes of the pinnule or its segments, oriented upward, nearly straight to the plane of the lamina (Figure 2A,B and Figure 3A–E). Involucres are tubular, slightly curved, immersed in lobes or segments, winged throughout, 1.0–2.2 mm long and 0.3–0.6 mm wide, ca. 3–5 times as long as wide (Figure 3 and Figure 4). Involucre mouths are usually not dilated (Figure 3C,E–G and Figure 4). Receptacles are filiform, long exserted, and up to 3.1 mm long (Figure 3B,C,F,G and Figure 4).

**Remarks:** Although Ebihara et al. [3] subdivided the *Trichomanes s.l.* into eight genera mainly based on the results of molecular phylogeny [3,13], it was virtually impossible to find macro-morphological characters that consistently discriminated these genera for all species [16]. It is especially difficult to put this classification into practice for the present fossils because they apparently lack their rhizome part: a very important character for generic identification. So, we adopted the concept of *Trichomanes s.l.* [17,18,19], which is still used today [16,41].

**Figure 5:**
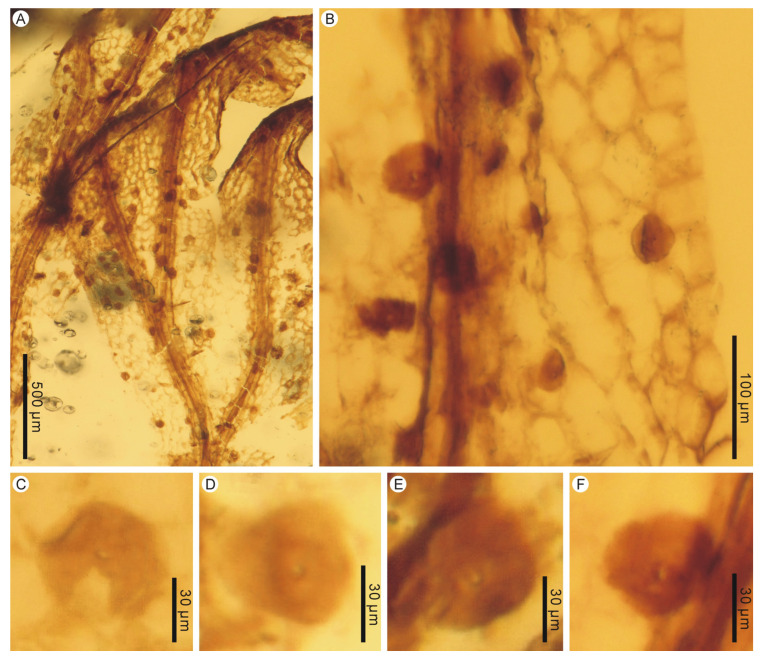
Associated pollen grains adhering on lamina surface of *Trichomanes angustum* comb. nov. PB201715a. (**A**) Lamina portion showing adhering pollen grains. (**B**) Enlargement of lamina showing adhering pollen grains. (**C**–**F**) Individual monoporate pollen grain.

It is strange that some pollen grains adhere on the lamina surface in PB201715 (Figure 5A,B), where filmy fern sporangia and spores are completely absent, although there are rich sori and involucres. These pollen grains are monoporate, with a round to rounded triangle, 31–65 μm in diameter (Figure 5C–F). Monoporate pollen is indeed present in some Mesozoic gymnosperm taxa, such as *Admolia* Batten and *Perinopollenites* Couper [42]. The former is an unknown gymnosperm reported from the Early Cretaceous of the UK and China, while the latter belongs to taxodioid Cupressaceae (‘Taxodiaceae’) and is widely distributed around the world during the Jurassic to the Cretaceous period [42]. However, the lack of ornamentation details hampers a definite identification.

## 4. Discussion

### 4.1. Comparisons

**Figure 6:**
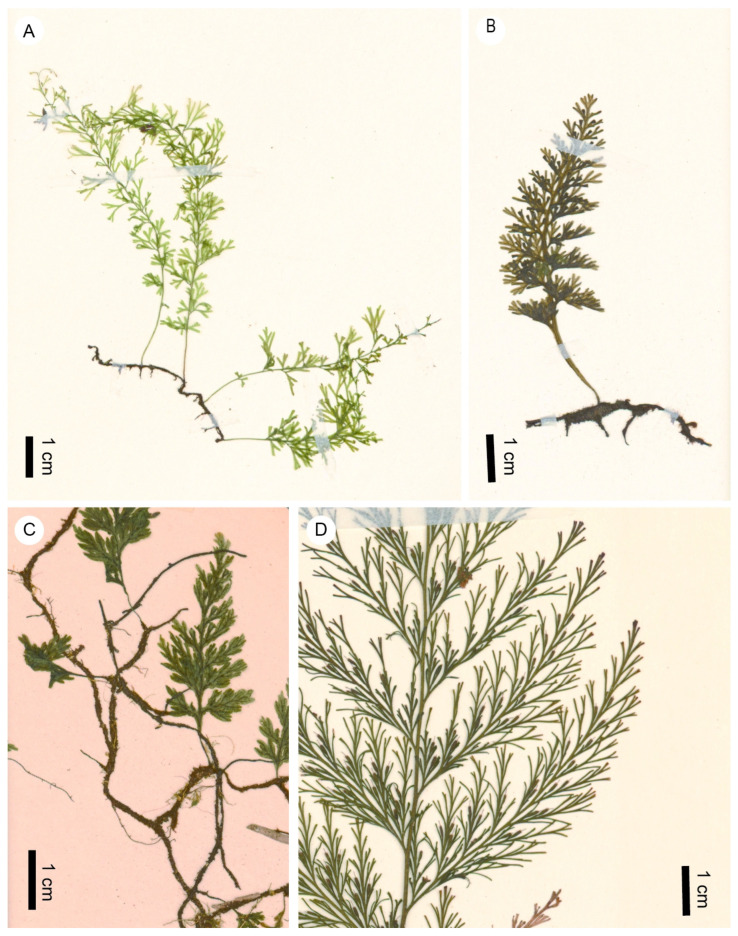
Examples of extant species of *Trichomanes s.l.* (**A**) *Polyphlebium angustatum* (Carmich.) Ebihara et Dubuisson (TNS VS-1310151, Brazil). (**B**) *Vandenboschia nipponica* (Nakai) Ebihara (TNS VS-1222341, Japan). (**C**) *Crepidomanes schmidtianum* (Zenker ex Taschner) K.Iwats. (subg. *Crepidomanes*) (TNS VS-1032081, Japan). (**D**) *Crepidomanes thysanostomum* (Makino) Ebihara et K.Iwats. (subg. *Nesopteris*) (TNS VS-9506554, Japan).

Although highly challenging, we made a great effort to narrow down this identification according to the classification of Ebihara et al. [3], who subdivided *Trichomanes s.l.* into eight genera, namely *Abrodictyum* C.Presl, *Callistopteris* Copel., *Cephalomanes* C.Presl, *Crepidomanes* C.Presl, *Didymoglossum* Desv., *Polyphlebium* Copel., *Trichomanes* L. *sensu stricto* (*s.str.*), and *Vandenboschia* Copel., each with one to four subgenera. Firstly, our fern remains could be distinguished from the Palaeotropical genera *Callistopteris* and *Cephalomanes* as well as the predominantly Neotropical genus *Trichomanes* s.str. by having tubular involucres, while the latter three genera had campanulate involucres [3,43]. In addition, our remains were divided at least tripinnate with one cell layer thick pinnae as well as anadromous venation, while *Cephalomanes* only has a once-pinnate lamina with asymmetric pinnae and *Trichomanes* s.str. also displayed other different features in its five subgenera. For example, once-pinnate to bi-pinnate-pinatifid lamina was found in subgenera *Afrotrichomanes* and *Lacostea*, more than one cell layer thick lamina was identified in subgenera *Davalliopsis* and *Feea*, and usually, catadromous venation is found in subgenus *Trichomanes* [3]. Secondly, our fern remains differ from *Abrodictyum* in having more than three rows of laminar cells between the midrib and margins and having straight internal cell walls, while the lamina of most *Abrodictyum* species is reduced to less than three rows of cells between the midrib and the margins and internal cell walls are wavy or pitted [3,14,44]. Finally, our fern remains could be confined to a hemiepiphytic/epiphytic clade within the trichomanoids, comprising *Polyphlebium*, *Didymoglossum*, *Crepidomanes,* and *Vandenboschia* [10]; however, the absence of false veinlets clearly differentiated our fern remains from *Didymoglossum* that always have false veinlets [3,14]. Therefore, we could only narrow down the identification of our fossils to three candidate genera, namely *Polyphlebium*, *Crepidomanes,* and *Vandenboschia*. Unfortunately, we have not found any other traits in our fossils that allow the clear-cut identification of these three genera by lamina characters alone (Figure 6). To identify the genus, we needed to add more evidence, such as rhizome and rachis characters.

*Polyphlebium* is a primarily pantropical genus with ca. 15 species, mainly distributed in the Southern Hemisphere [3,45,46,47,48]. The present fossils had more divided lamina than most species of *Polyphlebium,* except the two strictly Neotropical species, *P*. *angustatum* (Carmich.) Ebihara et Dubuisson (Figure 6A) and *P*. *capillaceum* (L.) Ebihara et Dubuisson, one endemic species, *P*. *exsectum* (Kunze) Ebihara et Dubuisson, in Chile, as well as a Central and South American species *P*. *hymenophylloides* (Bosch) Ebihara et Dubuisson. However, *P*. *capillaceum* has filiform linear ultimate lobes, and *P*. *hymenophylloides* has differentiated marginal elongate cells [48,49]. The present fossils look very similar to *P*. *angustatum* in gross morphology [48] (Figure 6A) but still differ from it in having extremely narrow ultimate lobes (usually 0.3–0.4 mm wide) and non-dilated involucre mouths. The ultimate lobes in *P*. *angustatum* are 0.5–0.7 wide [48].

*Crepidomanes* include two subgenera, *Crepidomanes* and *Nesopteris*, and ca. 30 species, distributed from Palaeotropics to northern temperate regions [3]. In addition, one species in the Neotropics (*C*. *pyxidiferum* (L.) Dubuisson et Ebihara) was found [50]. The subgenus *Crepidomanes* includes three sections and differs from our fern remains as follows. In section *Crepidomanes*, false veinlets are often present, and involucre mouths are usually bilabiate; section *Gonocormus* has campanulate involucre with dilate mouths; section *Crepidium* has double rows of elongate marginal cells [3]. It is to be noted that some members of section *Crepidomanes* lack false veinlets, e.g., *C*. *schmidtianum* (Zenker ex Taschner) K.Iwats. (Figure 6C) and *C*. *vitiense* (Baker) Bostock. The subgenus *Nesopteris* has rather large and more than 15 cm long fronds [3], but the relatively long involucres in our fossils reminded us of this subgenus (Figure 6D).

*Vandenboschia* is a pantropical genus with two subgenera, *Vandenboschia* and *Lacosteopsis*, and ca. 15 species that are often hemiepiphytic and occasionally terrestrial [3,10,46]. The subgenus *Vandenboschia* owns bipinnate or more finely divided lamina (Figure 6B), while the subgenus *Lacosteopsis* has once pinnate lamina [3]. Thus, the identification of the subgenus *Lacosteopsis* could be excluded. In addition, this genus always has minute clavate hairs on its rachises [3]. If our two large fossils (Figure 1A,B and Figure 2A,B) stem from the lamina portion rather than from single large lateral pinnae, the identification of subgenus *Vandenboschia* could also be excluded owing to its minute clavate hairs on rachises.

### 4.2. Palaeoecological Implications

Epiphytes are non-parasitic plants that germinate and grow on phorophytes at all stages of life [51]. Thus, epiphytes and their host phorophytes form a typical commensal relationship. Filmy ferns represent mostly a dense community of low epiphytes in the forest understory; however, some species can fully cover trunks and branches in cloud forests [44,52,53]. It is probable that the fossil species *Trichomanes angustum* was an epiphyte because it could be morphologically confined to a hemiepiphytic/epiphytic clade within the trichomanoids [10]. According to the analysis of two-gene (*rbcL* + *rps4*), the crown group of this hemiepiphytic/epiphytic clade started to evolve around 121.7 ± 12.3 Ma [10], which is in agreement with our assignation of *T*. *angustum* to this clade. The mid-Cretaceous Kachin amber forest was highly tropical and probably located close to the seashore [32,37,38]. It was rich in epiphytes that are known to be mainly composed of Porellalean leafy liverworts [54,55,56,57,58,59,60,61,62,63,64,65] and Dicranalean and Hypnodendralean mosses [66,67,68,69,70]. The discovery of *T*. *angustum,* along with *Hymenophyllites kachinensis* and *H*. *setosus* in the mid-Cretaceous Kachin amber [27], further enriches the species diversity of the epiphytic palaeocommunities.

There are still some intriguing aspects of *T*. *angustum* that remain unknown, including the host phorophytes. However, it is likely that *T*. *angustum* grew on resin-producing gymnosperm trees so that it was easily captured by the resin, and the monoporate pollen adhering on the fern lamina surface (Figure 5) represents pollen rain from the phorophyte trees or nearby trees. The mid-Cretaceous Kachin amber forest was dominated by various gymnosperm trees, including members of Araucariaceae and Cupressaceae [32,37]. Nuclear magnetic resonance spectroscopic studies and anatomical analyses of fossil wood fibers have indicated that araucarioid trees of the Araucariaceae, especially *Agathis* Salisb., were determined as the source of Kachin amber [71].

### 4.3. Paleobiogeographic History of Hymenophyllaceae

Copeland [72] suggested an Antarctic origin for the Hymenophyllaceae according to the fact that most filmy ferns grow in the Southern Hemisphere, with numerous monotypic groups in austral regions. Iwatsuki [9,73] believed that filmy ferns evolved in the tropics and subsequently dispersed from there. From the analysis of *rbcL* sequence data, Dubuisson et al. [74] inferred a basal position for Asian groups in *Trichomanes*, while the most basal taxa within *Hymenophyllum* are also Paleotropical and/or austral [75]. Thus, Dubuisson et al. [74] speculated that Hymenophyllaceae probably arose and first diverged in the Paleotropics, possibly in Asia. Divergence time estimates indicate that Hymenophyllaceae might have evolved during the Carboniferous to the Triassic period [2,5,6,10,20,43] and split into hymenophylloids and trichomanoids in the Middle Jurassic period [10]. Numerous Paleozoic and Mesozoic Hymenophyllaceae-like fronds were assigned to the extinct genera *Hymenophyllites*, *Trichomanides,* and *Trichomanites* [76,77,78,79] or to the extant genus *Hymenophyllum* [80], but the affinity of these fossils to Hymenophyllaceae is quite uncertain owing to the lack of definite evidence of a membranaceous habit or marginal indusiate sori [21,81].

**Figure 7:**
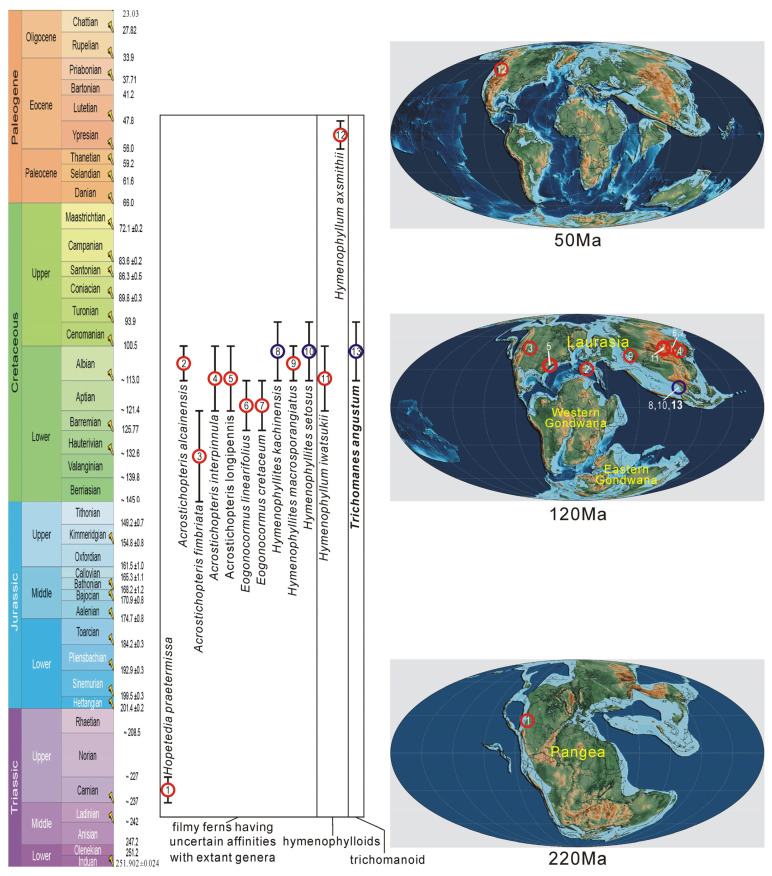
The temporal and spatial distribution of Hymenophyllaceae fossils in the world. Geologic time scale and palaeogeographic maps were from Cohen et al. [85] (updated, v. 2023/04) and Scotese [82].

The earliest reliable Hymenophyllaceae fossils are reported from the Late Triassic Pekin Formation of North Carolina, USA [21], followed by an apparent gap in the Jurassic period (Table 1; Figure 7). Hymenophyllaceae diversified in the Early to middle Cretaceous period of Laurasia with several fossil genera and species reported from Spain, Kazakhstan, Mongolia, China, and the USA [11,25,26,27,28,29,30,31] (Table 1; Figure 7). Some younger fossils of Hymenophyllaceae have been recently reported to be from the early Eocene of Okanogan Highlands, Washington, USA [24] (Table 1; Figure 7). It is strange that all unequivocal fossils of Hymenophyllaceae were reported from the Northern Hemisphere. Despite being a worldwide fern lineage predominantly distributed in tropical mountains and south-temperate areas, Hymenophyllaceae lack a definitive fossil record in the Southern Hemisphere (Figure 7). Although some putative fossils of Hymenophyllaceae were reported as *Trichomanides laxum* Tenison-Woods and *T*. *spinifolium* Tenison-Woods from the Jurassic of Queensland, Australia [76] and *Hymenophyllum priscum* Menéndez from the Late Cretaceous of Chile [80], the identification to Hymenophyllaceae has been thought to be wrong or less convincing [21]. Although the bulk of the extant diversity of Hymenophyllaceae appears to have accumulated later in angiosperm-dominated forests during the Cenozoic [10], the reliable fossil record of Hymenophyllaceae is scarce in the Cenozoic (Table 1; Figure 7).

All in all, present fossil evidence indicates a tropical Pangea origin for Hymenophyllaceae at the latest in the Triassic when all continents coalesced into a single landmass Pangea under a global hothouse climate [82] (Figure 7), and some notable diversity was already developed in low and middle latitude areas of Laurasia in the mid-Cretaceous when the earth experienced a hothouse climate again [82] (Figure 7).

**Table 1:** Reliable fossil record of Hymenophyllaceae.

Taxon	Age	Stratigraphic Horizon	Locality	Reference
*Acrostichopteris alcainensis* Sender	Middle–late Albian, Early Cretaceous	Upper Member, Escucha Formation	Alcaine village, Teruel Province, Spain	[31]
*Acrostichopteris fimbriata* Knowlton	late Berriasian–Barremian, Early Cretaceous	Kootanie Formation	Meridith mine, Cascade County, Montana, USA	[29]
*Acrostichopteris interpinnula* Meng et Chen	Aptian–Albian, Early Cretaceous	Fuxin Formation	Xinqiu coal mine, Fuxin, Liaoning, China	[30,83]
*Acrostichopteris longipennis* Fontaine	late Aptian–early Albian, Early Cretaceous	Potomac Group	Baltimore, Maryland, and Richmond, Virginia, USA	[28,31]
*Eogonocormus cretaceum* Deng	late Barremian–early Aptian, Early Cretaceous	Lower Coal-bearing Member, Huolinhe Formation	Huolinhe Basin, Inner Mongolia, China	[25,84]
*Eogonocormus linearifolius* Deng	late Barremian–early Aptian, Early Cretaceous	Lower Coal-bearing Member, Huolinhe Formation	Huolinhe Basin, Inner Mongolia, China	[25,84]
*Hopetedia praetermissa* Axsmith et al.	late Carnian, Late Triassic	Middle portion of Pekin Formation	Boren Clay Company pit near Gulf, North Carolina, USA	[21]
*Hymenophyllites kachinensis* Y.Li et Y.-D.Wang	Upper Albian–lower Cenomanian, mid-Cretaceous	No data	Amber mines near Tanai, Kachin State, Myanmar	[27]
*Hymenophyllites macrosporangiatus* Vachrameev	Middle Albian, Early Cretaceous	Kysylshen Formation	Karachetau and Kysylshen, western Kazakhstan	[26]
*Hymenophyllites setosus* Y.Li et Y.-D.Wang	Upper Albian–lower Cenomanian, mid-Cretaceous	No data	Amber mines near Tanai, Kachin State, Myanmar	[27]
*Hymenophyllum axsmithii* Pigg et al.	Ypresian, early Eocene	Tom Thumb Member, Klondike Mountain Formation	Boot Hill, Republic, Washington, USA	[24]
*Hymenophyllum iwatsukii* Herrera et al.	Aptian–Albian, Early Cretaceous	Tevshiin Govi and Khukhteeg Formations	Tevshiin Govi and Tugrug, central Mongolia	[11]
*Trichomanes angustum* comb. nov.	Upper Albian–lower Cenomanian, mid-Cretaceous	No data	Amber mines near Tanai, Kachin State, Myanmar	This paper

## 5. Conclusions

Here, we report the first definitive fossil species of *Trichomanes s.l.* as *T*. *angustum* comb. nov., which is combined from the previously described *Hymenophyllites angustus*, based on newly found fertile material from the mid-Cretaceous Kachin amber, Myanmar. Morphological comparisons suggest that *T*. *angustum* can be confined to a hemiepiphytic/epiphytic clade within the trichomanoid. Inferred to be an epiphytic fern, *T*. *angustum* further enriches the species diversity of the epiphytic palaeocommunities in mid-Cretaceous Kachin amber, mainly comprising Porellalean leafy liverworts and Dicranalean and Hypnodendralean mosses. Fossil records indicate that Hymenophyllaceae probably originated in the tropical Pangea at the latest, in the Triassic and accumulated some notable diversity in low and middle-latitude areas of Laurasia by the mid-Cretaceous.

## Data Availability

All data are reported in this paper.
